# Influence of Alkyl Chain Length on Thermal Properties, Structure, and Self-Diffusion Coefficients of Alkyltriethylammonium-Based Ionic Liquids

**DOI:** 10.3390/ijms22115935

**Published:** 2021-05-31

**Authors:** Roksana Markiewicz, Adam Klimaszyk, Marcin Jarek, Michał Taube, Patryk Florczak, Marek Kempka, Zbigniew Fojud, Stefan Jurga

**Affiliations:** 1 NanoBioMedical Centre, Adam Mickiewicz University, Wszechnicy Piastowskiej str. 3, 61-614 Poznań, Poland; marcin.jarek@amu.edu.pl (M.J.); patryk.florczak@amu.edu.pl (P.F.); marek.kempka@amu.edu.pl (M.K.); stjurga@amu.edu.pl (S.J.); 2 Department of Macromolecular Physics, Faculty of Physics, Adam Mickiewicz University, Uniwersytetu Poznańskiego str. 2, 61-614 Poznań, Poland; michal.taube@amu.edu.pl (M.T.); zbigniew.fojud@amu.edu.pl (Z.F.)

**Keywords:** ionic liquids, bis(trifluoromethanesulfonyl)imides, structure of ionic liquids, NMR diffusometry, small-angle X-ray scattering, SAXS, differential scanning calorimetry, DSC, infrared spectroscopy, FTIR

## Abstract

The application of ionic liquids (ILs) has grown enormously, from their use as simple solvents, catalysts, media in separation science, or electrolytes to that as task-specific, tunable molecular machines with appropriate properties. A thorough understanding of these properties and structure–property relationships is needed to fully exploit their potential, open new directions in IL-based research and, finally, properly implement the appropriate applications. In this work, we investigated the structure–properties relationships of a series of alkyltriethylammonium bis(trifluoromethanesulfonyl)imide [TEA-R][TFSI] ionic liquids in relation to their thermal behavior, structure organization, and self-diffusion coefficients in the bulk state using DSC, FT-IR, SAXS, and NMR diffusometry techniques. The phase transition temperatures were determined, indicating alkyl chain dependency. Fourier-transformed infrared spectroscopy studies revealed the structuration of the ionic liquids along with alkyl chain elongation. SAXS experiments clearly demonstrated the existence of polar/non-polar domains. The alkyl chain length influenced the expansion of the non-polar domains, leading to the expansion between cation heads in polar regions of the structured IL. ^1^H NMR self-diffusion coefficients indicated that alkyl chain elongation generally caused the lowering of the self-diffusion coefficients. Moreover, we show that the diffusion of anions and cations of ILs is similar, even though they vary in their size.

## 1. Introduction

The properties of ionic liquids (ILs), mainly their negligible volatility, thermal and electrochemical stability, wide liquid range, high conductivity, and ability to dissolve organic, inorganic, and polymeric compounds, have made them extremely popular in multiple applications. They are widely used as molecular solvents in the areas of synthesis and separation science, as media in catalysis, electrolytes, lubricants, and other task-specific materials [1,2]. ILs are used are not only in chemistry, physics, and materials science but, with the exploration of their biological activity, very often also in medicine, pharmacy, and agrisciences [3]. Importantly, their physicochemical properties, described as tunable and designable, need to be always properly recognized and defined. This tunability also comes with a great challenge, since the amount of theoretical ionic liquids that can be obtained is limited only by organic and inorganic chemistry [4].

A thorough understanding of their properties and structure is always needed to exploit their potential and fully implement their appropriate application. An important property to be considered is the ionic composition of ILs, which makes them primarily different from popular organic solvents and very valuable in applications in which their conductivity, viscosity, or behavior at the solid–liquid interface are important. Moreover, ILs possess a well-defined structure both in the bulk state and at interfaces, which is further strictly connected to their solvent capabilities [5,6]. Worth noticing is that ILs can participate in various interactions, such as van der Waals and Coulombic interactions, hydrogen bonds, and specific interactions, including hydrogen/halogen bonding. An important issue is also related to the length of the alkyl chain attached to ILs’ cation. Changes in this length varies ILs volume, mass, shape, and flexibility and, more importantly, their application properties [7].

Even though a vast number of works on the structure and properties of ionic liquids are present in the literature, experimental as well as theoretical studies are needed on this topic, especially considering those ILs that are less often used and more environmentally benign, e.g., ammonium, diazolium/triazolium, or nature-inspired ILs [4,8,9]. Two of the most widely explored groups of ILs are imidazolium and pyridinium [10,11]. Therefore, this work aims to present the influence of the alkyl chain length in the homologous series of alkyltriethylammonium bis(trifluoromethanesulfonyl)imide ([TEA-R][TFSI]) ionic liquids on their thermal behavior, structure organization, and other relevant specifics of the series. [TEA-R][TFSI] ILs are known to serve as electrolytes for lithium-ion battery applications, as media for anodic oxidation of organic pollutants, and as media of enantioselective or catalytic reactions [12,13,14,15]. To the best of our knowledge, a complete series of these ILs has never before been evaluated in terms of their thermal, structural, and macroscopic properties.

## 2. Results and Discussion

### 2.1. Thermal Properties

The thermal properties of ionic liquids are essential when it comes to their application, e.g., in supercapacitors, solar cells, batteries, impurities extraction, chemical reactions, and catalysis media [16]. The literature reports data about the thermal behavior of imidazolium and/or pyridinium ILs, usually obtained with the use of various methodologies (with various scanning rates, annealing periods, sample size, thermal history, and sample purity) [17]. To the best of our knowledge a complete series of alkyltriethylammonium bis(trifluoromethanesulfonyl)imides (C_4_–C_16_ alkyl chain length) has never before been examined thoroughly in terms of their molecular dynamics and thermal behavior. Phase transition temperatures and the associated enthalpies of transitions (obtained from the peak area of the appropriate transition) observed for the series of alkyltriethylammonium ILs are summarized in Table 1.

The results presented in Table 1 show sequentially the crystallization temperature (T_cryst_), which is defined as the onset of an exothermic peak on cooling from a liquid to a solid state, the glass-transition temperature (T_g_), which is the midpoint of a small heat capacity change on heating from the amorphous glass state to a liquid state, the cold crystallization temperature (T_cc_), which is defined as the onset of an exothermic change on heating from subcooled liquid state to a solid-state, the solid–solid transition temperature (T_s-s_), which is defined as the onset of a small endothermic change on heating, and the melting temperatures (T_m_), which are taken as the onset of an endothermic peak also on heating [18,19]. The thermograms of all ILs are presented in Supplementary Figures S1–S7.

Crystallization was observed for ionic liquids with shorter to medium alkyl chain lengths (4–8) and again for ionic liquids with longer alkyl chain lengths (14–16). [TEAC_10_][TFSI] and [TEAC_12_][TFSI] did not exhibit the crystallization change in the measured range, and from the calculations of associated enthalpies, one can notice that with the elongation of the alkyl chain, the enthalpies became smaller with the increase of the alkyl chain from 4 to 8. Crystallization was not observed within the measured range for octyl and decyl alkyl chains; the crystallization enthalpies increased with further alkyl chain elongation. Moreover, ionic liquids with butyl, dodecyl, and tetradecyl substituents underwent cold crystallization in the heating mode.

Ionic liquids with butyl, tetradecyl, and hexadecyl substituents ([TEAC_4_], [TEAC_14_] and [TEAC_16_]) did not exhibit glass transition in the region above 173.15 K. The glass transition temperatures for the series comprising 6 to 12 carbon atoms shifted to higher values with the elongation of the alkyl chain in the quaternary ammonium cation, i.e., from 185.70 K for TEAC_6_ to 201.77 K for TEAC_12_. The data presented here show good agreement with the available literature values [19].

The literature also describes a “2/3 golden rule”, which means that for all compounds, that ratio of the temperatures of glass transition and melting (T_g_/T_m_) is around 0.66 [20]. Lately, it was shown that for aprotic ionic liquids, the usual value of the ratio is 0.75 [17]. In the case of the [TEA-R] series, such values could be determined only for two ILs—[TEAC_10_][TFSI] and [TEAC_12_][TFSI]—because they were the only ones for which both melting point and glass transition temperatures were exhibited. For both ILs, the ratio is equal to 0.73. Similarly to other examples found in the literature of alkyl homologous series of ionic liquids, the melting temperature was determined for short alkyl chain (butyl), not observed for hexyl and octyl substituents, and, with further chain elongation, shown to increase (with a maximum for the [TEAC_14_][TFSI]). Moreover, ILs with butyl, dodecyl, tetradecyl, and hexadecyl substituents underwent a weak solid–solid transition related to a structural change in solid phase, at the temperatures of 236.54, 264.42, 278.61, and 276.66 K, respectively. We also noticed that for [TEAC_16_][TFSI], in addition to melting and solid–solid transition observed in the heating range, another solid–solid transition occurred right before melting (Figure S7).

The influence of the heating rate on the thermal properties was also examined using rates of 10, 5 and 2 K min^−1^. As an example, [TEAC_12_][TFSI] was taken into consideration, since it presented more evident changes. The temperatures of phase transitions measured for dodecyltriethylammonium bis(trifluoromethanesulfonyl)imide for three temperature rates are presented in Table 2.

As for the melting temperature, the shift was negligible, but for other transitions, it was more evident. The glass transition temperature T_g_ shifted at a faster heating rate to higher temperature values. For cold crystallization and solid–solid transition, we observed a change to lower values between 2 and 5 K min^−1^, yet at the rate of 10 K min^−1^, we observed even higher values. According to the literature, such changes might be a result of nanostructured organization at a molecular level [4,20,21].

### 2.2. Infrared Spectroscopy

Vibrational spectroscopy is a powerful tool allowing the characterization and understanding not only of the structure of ILs but also of the nature of ionic interactions, anion-cation hydrogen bonds, and molecular conformations [6]. In the case of bis(trifluoromethanesulfonyl)imide anion, ILs with 1-alkyl-3-methylimidazolium and dialkylpyrrolidinium cations are the most widely studied and described in the literature. Therefore, we have decided to study and discuss the FT-IR spectra of a alkyltriethylammonium homologous IL series. The spectra of the prepared ionic liquids are presented in Supplementary Materials (Figure S8).

**Figure 1:**
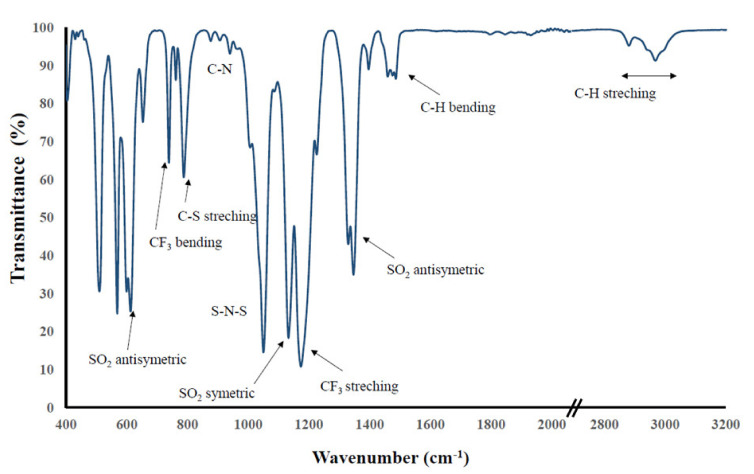
FT-IR spectra of butyltriethylammonium bis(trifluoromethanesulfonyl)imide [TEA-C4][TFSI].

Figure 1 presents the spectra of a representative IL, [TEA-C4][TFSI], where the most important absorption bands derived from various groups of both the butyltriethylammonium cation and the bis(trifluoromethanesulfonyl)imide anion are marked.

The FT-IR spectra of the presented ILs are dominated by bands derived from anion vibrations. The main bands are the shuttle band derived from bending and stretching of S–N–S bonds around 650 and 750 cm^−1^, respectively, sulfur–nitrogen vibrations about 1050, SO_2_ symmetric and antisymmetric vibrations around 1120 and 620 with 1350 cm^−1^, respectively, and bending and stretching vibrations of CF_3_ at 570 and 1150 cm^−1^. The bis(trifluoromethanesulfonyl)imide anion was the subject of many vibrational spectroscopy studies because it has been extensively used in polymer electrolytes [6,22,23]. Information regarding not only the inorganic salts but also other ionic liquids with this anion have been widely discussed in the literature.

Importantly, the vibrational frequencies of the anion are influenced by the polarizing power of the cation of the chosen IL; nevertheless, the change in the alkyl length does not influence the [TFSI] shifts. The main cation shifts visible in the spectra are those originating from the C–N bond around 850 to 1000 cm^−1^, C–H bending vibrations around 1500 cm^−1^, and C–H stretching vibrations of the alkyl chains at 2800–3000 cm^−1^.

**Figure 2:**
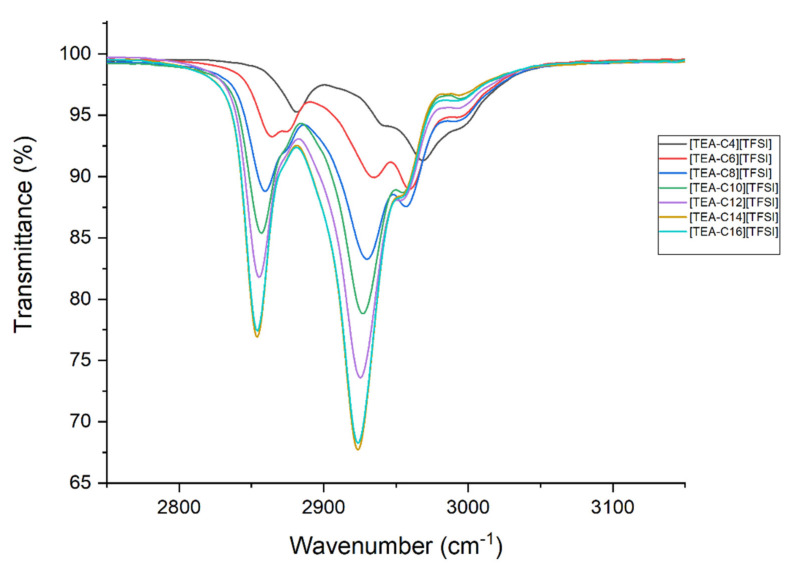
Stretching vibrations of [TEA-R][TFSI].

When analyzing the anion, no significant change could be found within the series, as also observed for other [TFSI]-based homologous series of ILs. The structurization of ILs, however, could be observed through a thorough analysis of the shifts visible in the region from 2800 to 3000 cm^−1^, as presented in Figure 2.

In the range of 2800–3200 cm^−1^, a complex pattern of overlapped bands derived from several C–H stretching modes were visible for [TEA-R][TFSI]. The elongation of the alkyl chain length caused a substantial effect on those vibrations. It can be noticed that, as the length of the alkyl chain increased, there was a shift towards shorter wavelengths, accordingly increasing the intensity of the signals. Lowering C–H stretching frequencies of the symmetric and asymmetric vibrations of the CH_2_ and CH_3_ groups are presented in Table 3. Such shifts are suggested to be caused by the aggregation of the –CH_2_ groups in the alkyl chain, driven by van der Waals forces [23]. Moreover, the elongation of the alkyl chain to 16 carbon atoms did not cause further shift change in the vibration spectra, only lowered the intensity. This might be explained by the fact that longer alkyl chains cause chain curling. Such behavior is well known for all quaternary ammonium salts [1,4,24,25].

### 2.3. Small-Angle X-ray Scattering

Small-angle, along with wide-angle X-ray scattering, is used for investigation of ILs structure in bulk as well as in a form of mixtures. The major impact of the length of the alkyl chain on IL structure relates to the formation of an amphiphilic nanostructure. For appropriately long alkyl chains, segregation between polar and apolar domains occurs, which leads to the creation of locally smectic or bicontinuous liquid structure [5].

**Figure 3:**
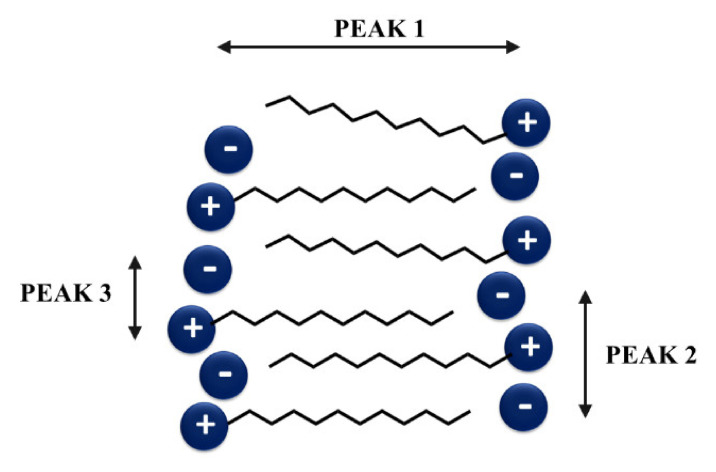
Schematic drawing of three peaks commonly observed in the SAXS patterns adapted from [26,27].

Ionic liquids with an appropriately long alkyl chain also exhibit three main peaks in small-angle X-ray scattering patterns (peak I, II, and III), which are assigned to specific correlations, demonstrating the occurrence of nm-scale segregation of the apolar side alkyl tails in a charged environment composed of the polar heads of cations and anions. A schematic representation of these correlation peaks is presented in Figure 3.

**Figure 4:**
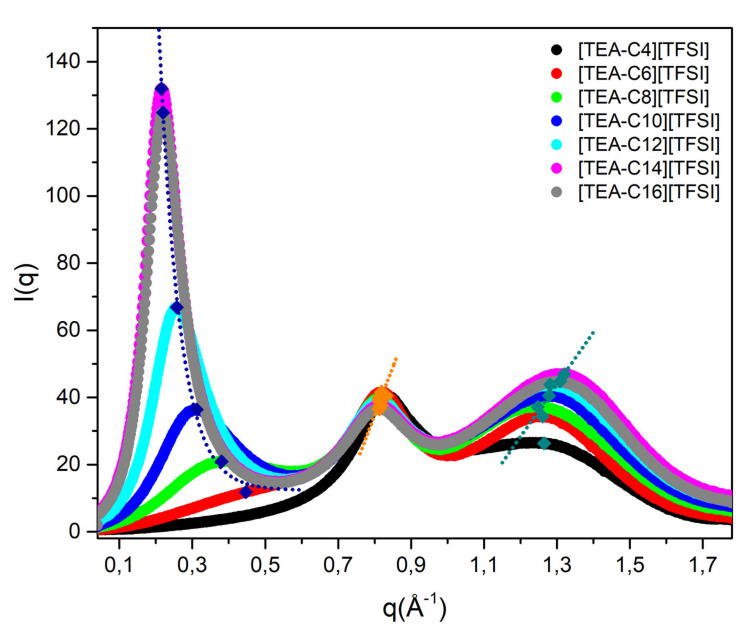
SAXS patterns of the homologous series [TEA-R][TFSI].

Peak *I* represents the correlation distance along the axis of alkyl chains, peak *II* corresponds to the same charge inter-ion distance (cation–cation and anion–anion) within the IL nanostructured network, and peak *III* represents the cation–anion distance (short-range correlations) [26]. The results of the performed SAXS experiments for the [TEA-R][TFSI] series presented as the peak intensity against the scattering vector are shown in Figure 4.

In the case of the prepared homologous series, it is obvious that nanostructure formation took place. It was most visible for alkyl chains with n > 6, which indicates that mesoscopic segregation was not present in the case of [TEAC_4_][TFSI]. The first peak which was correlated to the distance between layers in our case precisely showed that with the elongation of the alkyl chain, the distance between the layers increased. The non-polar character became more apparent, as the carbohydrate ratio increased. In the case of [TEAC_16_][TFSI], the distance remained at the same level as that for [TEAC_14_][TFSI], which might be connected to chain curling, similarly to what observed in the FT-IR spectra, as explained earlier. When peaks *II* and *III* are taken into consideration, the distance change was neglectable, which means that the same charge inter-ion, as well as the cation–anion distance, remained unchanged by the structural organization of ILs’ phases. Importantly, the nanostructures created by ILs are analogous to a surfactant mesophase [1,5]. Appropriate distance values are presented in Table 4. A change in the distance for the homologous series is also shown in Figure S9.

The nanostructured domains present in the [TEA-R][TFSI] series, as well as in other ionic liquids, have crucial impact on various properties, especially viscosity and density, but also on conductivity, since this property is connected with ILs polarity (polar domains). A variety of ionic liquids and their mixtures with solvents and other ILs were investigated via SAXS analysis. In the case of the homologous series of imidazolium ILs, namely, 1-alkyl-3methylimidazolium bis(trifluoromethanesulfonyl)imides [C_n_mim][TFSI], three peaks were also observed in the range of 0.2–0.6, ~0.9, and ~1.5 Å^−1^. When compared to the values obtained for the [TEA-R][TFSI] series, it is obvious that both cation–cation and anion–anion distances are shorter in the case of imidazolium ILs. Moreover, for the same cations, the literature also indicates that the appropriate distance changes along with anion exchange [28].

### 2.4. Nuclear Magnetic Resonance (NMR) Diffusion

NMR, used for studying translational diffusion, is an effective method for examining the interactions between ions in complex systems, which are characterized by chemical composition diversity and are observed in a wide range of compounds, including ionic liquids [29,30]. Moreover, due to the possibility of NMR experiment modifications, it is possible to investigate local and translational mobilities of cations and anions in ILs [31,32,33,34,35]. ^1^H and ^19^F are the most common nuclei used to measure the diffusion of ions in ILs, with nuclei such as ^13^C and ^31^P also being helpful [35,36,37]. Nuclear Magnetic Resonance Pulsed Gradient Spin-Echo (PGSE NMR) experiments allow for a precise determination of self-diffusion coefficients [38].

The PGSE pulse sequence utilizes well-defined magnetic field gradient pulses to label spins in a system during defined time intervals. A π/2 pulse is applied, which rotates a macroscopic magnetization from the *Z*-axis to the X–Y plane (i.e., perpendicular to the static field). During the first period *τ* at time *t*_1_, a gradient pulse with *δ* duration and magnitude *g* is applied, which at the end of the first period *τ*, causes a phase shift of spins. At the end of the first *τ* period, the π pulse is applied, which changes the sign of the phase shift from the first gradient pulse. The restoration of the precession signal is governed by the second gradient pulse of equal magnitude and duration. Suppose the spins do not undergo translational displacements along the *Z*-axis. In that case, gradient pulses have no effect, the phase of the nuclear spin is fully restored, and the spin-echo recorded at full amplitude. However, if the spins have moved, the overall phase loss and spin-echo amplitude are proportional to the diffusion coefficient, the gradient integral (δ*g), diffusion time Δ (interval between gradient pulses) [39,40]. The self-diffusion coefficient, *D*, can be easily obtained. Moreover, a thorough analysis of viscosity and molecular size can also be performed. Temperature measurements, on the other hand, are useful for the calculation of some thermodynamic parameters.

**Figure 5:**
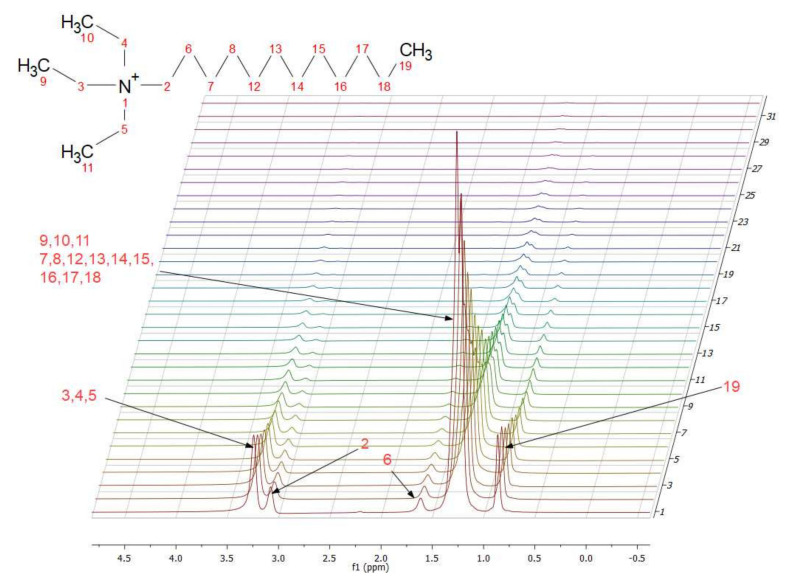
Example of stacked ^1^H NMR spectra for [TEAC_12_][TFSI]. Arrowed peaks correspond to characteristic chemical shifts for ILs, and their values are (from left to right): 3.24, 3.08, 1.61, 1.24, 0.86 ppm.

The diffusion decay of the spin-echo *M(g)* can be described by the Stejskal–Tanner equation as follows:

(1) $$A\left(g\right)=\frac{M\left(g\right)}{M_{g=0}}=A_{0}exp\left(-D\gamma^{2}\delta^{2}g^{2}\left(\Delta -\delta /3\right)\right)$$

where *D* is the diffusion coefficient, *γ* the magnetogyric ratio for the studied nuclei, *δ* the diffusion gradient length, *g* a field gradient amplitude, and Δ the diffusion time [41]. As the PGSE NMR technique is grounded on the analysis of the decay of appropriate NMR signals due to magnetic nuclei phase change caused by their translational displacement, a representative result of diffusion measurement using the PGSE ^1^H NMR technique for [TEAC_12_][TFSI] stacked in one layer is presented in Figure 5.

**Figure 6:**
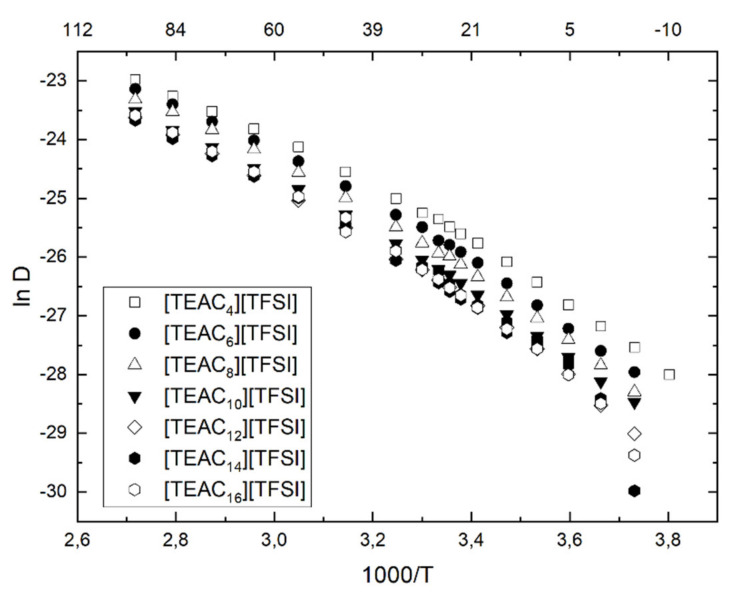
Diffusion coefficients measured by PGSE NMR in a temperature range from −5 to 95 °C.

It is possible to see some similarities and differences between particular ILs in the presented homologous series, as shown in Figure 6. Even though all decays were single-exponential (one-component diffusion over the examined temperature range was observed), one can see that the diffusion coefficients behaved similarly for the ILs. It was already proved that the diffusion coefficient values shift in the case of homologous series of ILs is not explained only by the size of the ions or the existing electrostatic interactions, but the explanation lies also in the ability of ionic liquids to form aggregates, clusters, or lamellar structures.

As the temperature lowered, a decrease in the diffusion coefficient could be observed. Importantly, similarly to other ionic liquids, e.g., imidazolium ones, the diffusion coefficient plots were not linear, but slightly curved down along with the temperature change. This implicates that the correlation time for the translational diffusion cannot be described by a simple Arrhenius thermally activated form [10]. Moreover, a decrease in the value of the diffusion coefficient was observed with the increase of the chain length, which corresponded to increasing the molecular volume of the ionic liquid [42]. Furthermore, chain elongation from one side increased the molecular volume of the liquids. On the other hand, the alkyl chain is more flexible. The trend visible at lower temperatures was traced back to partial crystallization, as visible in the DSC thermograms. The exact values of the diffusion coefficients are presented in Table S1.

For each IL in the entire temperature range measured, a similar trend could be seen, indicating that along with alkyl chain elongation, lowering of the diffusion coefficient was observed. A slight difference was visible for [TEAC_16_][TFSI], whose diffusion coefficients were higher than those of [TEAC_14_][TFSI].

There is a large body of published works where the diffusion of ions was measured in bulk ILs. In a series of works, diverse ILs were studied. Unique diffusion coefficients were obtained (for most of them), as well as for some other macroscopic physicochemical transport properties such as viscosity and conductivity [33,43,44,45]. For two selected ILs based on tetrafluoroborate anions and either [emim] or [bmim] cations, Hayamazu et al. have reported on local and translational molecular motions of cations and anions [46]. Other popular ionic liquid with methylimidazolium cations and bis(trifluoromethanesulfonyl)imide or bis(fluorosulfonyl)amide anions were studied by NMR techniques to investigate their rotational and translational motions and their corresponding binary systems with lithium salts [47]. It was mentioned that the molecular size of each type of ions has no direct impact on the diffusion coefficients [43]. In all those cases, the one-exponential behavior of diffusion was observed in a wide temperature range, from −20 to +60 °C [42].

**Figure 7:**
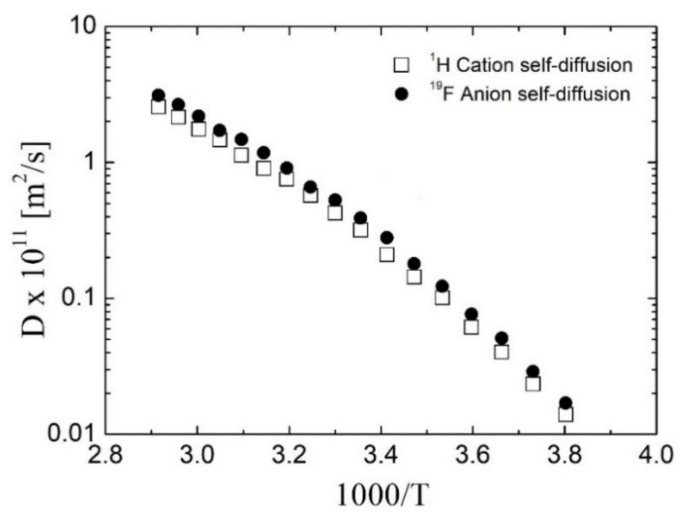
Diffusion coefficient measured by ^1^H and ^19^F PGSE NMR for [TEAC_12_][TFSI].

Ionic liquids are compounds in which at least two diffusion species can be always found (mainly cation and anion, individually). Since the ions control the most important properties (for example conductivity and solvation properties), particular consideration is being paid to individual ILs’ components. Figure 7 shows representative ^1^H and ^19^F diffusion coefficients of [TEAC_12_][TFSI]. The homologous series of [TEA-R][TFSI] exhibits a behavior similar to that of other ILs, meaning that the diffusion coefficients of ILs anion and cation, are similar, despite the difference in their size.

For glass-forming liquids, a generally accepted quantitative description of the temperature dependence of the diffusion rate does not exist. While the Arrhenius plot for diffusion temperature dependences in ILs is valid only in limited cases [48], empirical approaches such as the Vogel–Tamman–Fulcher (VTF) equation can be employed:

(2) $$D=D_{0}e^{\left(\frac{-kT_{0}}{T-T_{0}}\right)}$$

where the constants *D*_0_, *k*, and *T*_0_ are adjustable parameters [43]. The VFT relation usually fits to a variety of ionic liquids (imidazolium, piperidinium, and ammonium-based ILs) [10,42,49,50]. By fitting the temperature dependence to an Arrhenius behavior, we estimated the activation energies (*E_a_*) for cation diffusion, as presented in the Table 5. Fitting curves of each [TEA-R][TFSI] series can be found in Supplementary Materials.

The activation energy values exhibited nonmonotonous dependence along with the alkyl chain length change. For the presented series, the *E_a_* of ILs with shorter alkyl chains oscillated around 9 kJ mol^−1^, reaching ~11 kJ mol^−1^ for [TEAC_10_][TFSI], which means it is the most energy-demanding liquid in this homologous series when it comes to transitional motions. It is expected that the activation energy should increase along with alkyl chain elongation. It should be noted, however, that in the case of ILs, for which we deal not only with electrostatic interactions, but also with nanostructurization, with longer alkyl chains, shielding of these interactions and the formation of additional polar non-polar domains or the structure of ionic liquids may occur; we can talk about enabling an easier translation, which translates into a decrease in activation energy, as visible for alkyl chains from 10 to 14 carbon atoms, which is also observed in the literature [51]. On the other hand, further chain elongation caused the steric hindrance to be dominant again; therefore, we observed further *E_a_* increase.

## 3. Materials and Methods

### 3.1. Materials

Triethylamine, alkyl bromides (from 4 to 16 carbon atoms in the alkyl chain), lithium trifluoromethylsufonylimide, as well as all the solvents were purchased from commercial suppliers (Merck KGaA, Darmstadt, Germany, Avantor Performance Materials Poland S.A., Gliwice, Poland, Acros Organics B.V.B.A., a part of Thermo Fisher Scientific India Pvt. Ltd., Delphi, India) and used without further purification. Water purified with Mili-Q^®^ system (Merck Millipore, Merck KGaA, Darmstadt, Germany) was used for all applications (resistivity 18 MΩ cm^−1^).

### 3.2. Preparation of [TEA-R][TFSI] Ionic Liquids

**Figure 8:**
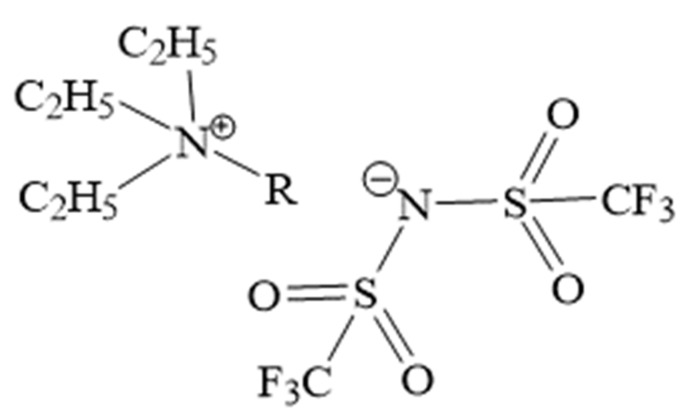
Structure of the obtained series of ILs (R stands for alkyl chain: C_2_H_5_, C_4_H_9_, C_6_H_13_, C_8_H_17_, C_10_H_21_, C_12_H_25_, C_14_H_29_ and C_16_H_33_).

We prepared ILs according to well-known literature protocols of (1) quaternization reaction and (2) ion-exchange reaction [52,53]. Each of the synthesized ILs was liquid at room temperature. A specific description of the preparation is described in the Supplementary Information. Before use, each of the obtained ILs was dried for 48 h in a vacuum desiccator. The samples for the measurements were prepared under an inert atmosphere (argon) in order to prevent moisture caption. The structure of each prepared ILs was confirmed via ^1^H (Agilent NMR 600 MHz, Agilent, Santa Clara, CA, USA) and ^13^C NMR (Agilent NMR 800 MHz, Agilent, Santa Clara, CA, USA) spectra analysis, available in Supplementary Materials. The structure of the homologous series of [TEA-R][TFSI] is presented in Figure 8.

### 3.3. Differential Scanning Calorimetry

Phase transition temperatures and the associated transition enthalpies of the [TEA-R][TFSI] homologous series of ILs were determined by differential scanning calorimetry, with a DSC 8000 (Perkin Elmer, Waltham, MA, USA) unit. Samples between 5 and 15 mg were placed in hermetic aluminum pans and cooled with a liquid-nitrogen cooling accessory to 173.15 K. Thermograms were recorded at three temperature heating/cooling rates of 10, 5, and 2 K min^−1^. The first step was always a 15 min isothermal step at 393.15 K, after which three cycles of cooling/heating were performed at a rate of 10 and 5 K min^−1^, and one cooling/heating cycle at 2 K min^−1^. The calibration of the DSC unit was performed as a multi-step process which involved baseline optimization, sample temperature calibration, furnace, and heat flow control. Indium and lead were used as a reference for calibration of temperature and enthalpy.

### 3.4. Infrared Spectroscopy

Fourier transform infrared spectroscopy was performed to characterize the structure of the homologous series of the prepared [TEA-R][TFSI] ionic liquids. Spectra were collected with a FT/IR-4700 (JASCO, Tokyo, Japan) spectrometer in the range of 400 to 4000 cm^−1^ at room temperature.

### 3.5. Small-Angle X-ray Scattering (SAXS)

Small-angle X-ray scattering studies were performed using the Xeuss 2.0 SAXS/WAXS system (XENOCS SAS, Grenoble, France) situated at the Joint Laboratory for SAXS studies at the Faculty of Physics, AMU. The system was equipped with MetalJet D2 microfocus X-ray source with a liquid metal (gallium) target (Excillum AB, Kista, Sweden) producing X-ray radiation at wavelength λ = 0.134 nm. SAXS data were recorded using PILATUS 3R 1M hybrid photon counting detector with an active sensor area (width × height) of 169 × 179 mm^2^ (DECTRIS AG, Baden, Switzerland). Samples were measured at room temperature in quartz capillaries. The background effect was eliminated through additional measurement of an empty capillary tube and subsequent subtraction of the obtained value. The real space correlation distance was calculated using the formula d = 2π/q, where q is a scattering vector ($q=\frac{4\pi }{\lambda }\sin\left(\theta \right)),$ 2θ is the angle between the incident and the scattered radiation, and λ is an X-ray wavelength.

### 3.6. PGSE NMR Diffusion

**Figure 9:**
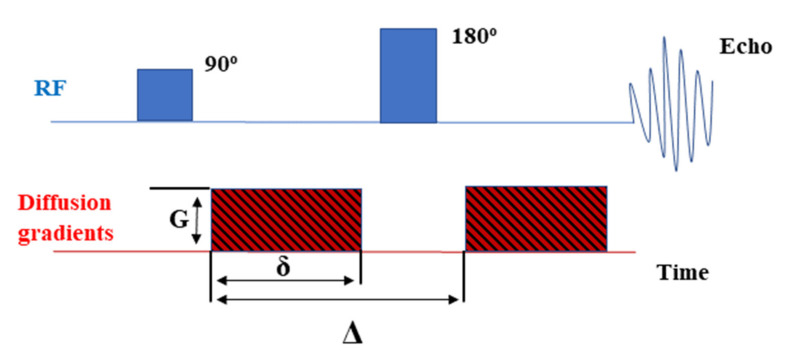
PGSE NMR sequence scheme.

The NMR study of the diffusion coefficients was performed with a 14 T Agilent NMR spectrometer (Agilent, Santa Clara, CA, USA), using a DOTY DSI−1372 gradient probe, g_z_ = 28 T/m with PGSE (Pulsed Gradient Spin-Echo) sequence scheme presented in Figure 9. The measurements were performed in the temperature range from −5 to 95 °C.

The samples were placed in a standard 5 mm external diameter NMR glass tube and heated stepwise by 5 degrees from −5 to 35 °C, and by 10 degrees in the range from 35 to 95 °C. Temperature-dependent self-diffusion coefficients and appropriate fits for each IL of the [TEA-R][TFSI] series is presents in Figure S10.

## 4. Conclusions

In this work, we presented the influence of alkyl chain length in the homologous series of alkyltriethylammonium bis(trifluoromethanesulfonyl)imide ionic liquids ([TEA-R][TFSI]) on their thermal properties, nanostructured organization, and self-diffusion coefficients. The thermal properties of the series of ionic liquids were described, indicating the existence of glass transition, crystallization, cold crystallization, and solid–solid transition and determining the melting temperatures. Solid–solid transition which was visible right before melting, indicated nanostructuring of the prepared ILs at the molecular level. Infrared spectroscopy showed the structuring of alkyl chains, as visible in the 2800–300 cm^−1^ region, responsible for C–H stretching vibrations of the alkyls. SAXS experiments clearly showed the existence of polar/non-polar domains. The alkyl chain length influenced the expansion of the non-polar domains, indicating the expansion between cation heads in polar regions of the structured IL. Self-diffusion coefficients of a series of bis(trifluoromethylsulfonyl)imide-based ILs were investigated in a wide temperature range. ^1^H self-diffusion coefficient exhibited single-component decays, and alkyl chain elongation generally caused lower self-diffusion values. Moreover, it was presented that the diffusion of anions and cations of IL are similar, even though they vary in their size.

## Figures and Tables

**Table 1:** Glass transition temperature (T_g_), crystallization temperature (T_cryst_) and enthalpy (ΔH_cryst_), cold crystallization temperature (T_cc_) and enthalpy (ΔH_cc_), solid–solid transition temperature (T_s-s_) and enthalpy (ΔH_s-s_), melting temperature (T_m_) and enthalpy (ΔH_m_) measured at the heating/cooling rate of 10 K min^−1^.

IONIC LIQUID	T_cryst_ [K], (ΔH_cryst_ [kJ mol^−1^])	T_g_ [K]	T_cc_ [K], (ΔH_cc_ [kJ mol^−1^])	T_s-s_ [K], (ΔH_s-s_ [kJ mol^−1^])	T_m_ [K], (ΔH_m_ [kJ mol^−1^])
[TEAC_4_][TFSI]	229.89 (−10.27)	–	208.64 (−2.25)	236.54 (2.92)	289.10 (17.36)
[TEAC_6_][TFSI]	228.18 (−1.10)	185.70	– –	– –	– –
[TEAC_8_][TFSI]	231.83 (−0.53)	192.00	– –	– –	– –
[TEAC_10_][TFSI]	– –	196.44	– –	– –	267.54 (0.45)
[TEAC_12_][TFSI]	– –	201.77	233.65 (−17.93)	264.42 (2.46)	278.20 (21.06)
[TEAC_14_][TFSI]	240.03 (−21.05)	– –	253.44 (−3.46)	278.61 (3.37)	294.50 (30.34)
[TEAC_16_][TFSI]	261.69 (−30.42)	– –	– –	276.66 (2.77)	289.41 (27.70)

**Table 2:** Glass transition temperatures (T_g_), crystallization temperatures (T_cryst_), cold crystallization temperatures (T_cc_), solid–solid transition temperatures (T_s-s_), melting temperature (T_m_) measured at the heating/cooling rate of 10 K min^−1^.

Transition, [K]	Heating Rate, [K min^−1^]		
	2	5	10
T_g_	100.13	100.54	101.77
T_cc_	129.37	128.01	133.65
T_s-s_	165.75	164.11	167.02
T_m_	178.75	178.86	178.20

**Table 3:** Frequency values of stretching vibrations of alkyl chains in [TEA-R][TFSI].

IL	Frequency Values [cm^−1^]			
	ν_sym_CH_2_	ν_sym_CH_3_	ν_asym_CH_2_	ν_asym_CH_3_
[TEAC_4_][TFSI]	2881	2949	2968	2989
[TEAC_6_][TFSI]	2863	2935	2959	2992
[TEAC_8_][TFSI]	2858	2930	2956	2990
[TEAC_10_][TFSI]	2857	2927	2946	2993
[TEAC_12_][TFSI]	2855	2925	2946	2993
[TEAC_14_][TFSI]	2854	2923	2946	2994
[TEAC_16_][TFSI]	2854	2923	2946	2991

**Table 4:** Frequency values of stretching vibrations of alkyl chains in [TEA-R][TFSI].

	Peak I [Å^−1^] (d [Å])	Peak I [arb.u.]	Peak II [Å^−1^] (d [Å])	Peak II [arb.u.]	Peak III [Å^−1^] (d [Å])	Peak III [arb.u.]
[TEAC_4_][TFSI]	-	-	0.84 (7.5)	48.20	1.25 (5.0)	30.74
[TEAC_6_][TFSI]	0.43 (14.6)	11.87	0.82 (7.7)	43.47	1.26 (5.0)	37.14
[TEAC_8_][TFSI]	0.40 (15.7)	22.79	0.82 (7.7)	42.74	1.24 (5.1)	40.60
[TEAC_10_][TFSI]	0.31 (20.3)	39.61	0.82 (7.7)	42.78	1.28 (4.9)	44.89
[TEAC_12_][TFSI]	0.26 (24.2)	71.78	0.82 (7.7)	42.47	1.29 (4.9)	48.23
[TEAC_14_][TFSI]	0.22 (28.6)	131.99	0.82 (7.7)	37.66	1.31 (4.8)	46.97
[TEAC_16_][TFSI]	0.22 (28.6)	131.19	0.81 (7.8)	38.84	1.31 (4.8)	48.15

**Table 5:** Activation energies of the [TEA-R][TFSI] series.

Cation:	[TEAC_4_]	[TEAC_6_]	[TEAC_8_]	[TEAC_10_]	[TEAC_12_]	[TEAC_14_]	[TEAC_16_]
E_a_ [kJ mol^−1^]	8.65	9.09	9.00	1 Please check if the original meaning is retained 1.17	9.47	5.28	7.58

## Supplementary Materials

The following are available online at https://www.mdpi.com/article/10.3390/ijms22115935/s1.
