# Supplementary material for: Influence of Alkyl Chain Length on Thermal Properties, Structure, and Self-Diffusion Coefficients of Alkyltriethylammonium-Based Ionic Liquids
Source: Int J Mol Sci. 2021 May 31;22(11):5935. doi: 10.3390/ijms22115935 (PMC8198313; doi:10.3390/ijms22115935)
Supplement: Supplementary file 1 [file ijms-22-05935-s001.zip › ijms-1231837-supplementary.pdf]

Supplementary materials:

Influence of the alkyl chain length on thermal properties, structure and NMR self-diffusion coefficients of alkyltriethylammonium-based ionic liquids

Roksana Markiewicz[1,*⊥], Adam Klimaszyk[1,2, ⊥], Marcin Jarek[1], Michał Taube[2], Patryk Florczak[1], Marek Kempka[1], Zbigniew Fojud[2], Stefan Jurga[1],

[1] NanoBioMedical Centre, Adam Mickiewicz University, Poznań, Wszechnicy Piastowskiej str. 3, 61-614 Poznań, Poland; email: roksana.markiewicz@amu.edu.pl

[2] Department of Macromolecular Physics, Faculty of Physics, Adam Mickiewicz University, Poznań, Uniwersytetu Poznańskiego str. 2, 61-614 Poznań, Poland

[⊥] The authors contributed equally.

Table of Contents:

I. Synthesis description and NMR Spectra of the prepared homologous series of alkyltriethylammonium bis(trifluoromethanesulfonyl)imides
II. Additional Figures
   a. Figure S1. DSC thermogram of butyltriethylammonium bis(trifluoromethylsulfonyl)imide [TEA-C4][TFSI] collected in the heating/cooling rate of 10 K min^{-1}.
   b. Figure S2. DSC thermogram of triethylhexylammonium bis(trifluoromethylsulfonyl)imide [TEA-C6][TFSI] collected in the heating/cooling rate of 10 K min-1.
   c. Figure S2. DSC thermogram of triethyloctylammonium bis(trifluoromethylsulfonyl)imide [TEA-C8][TFSI] collected in the heating/cooling rate of 10 K min^{-1}.
   d. Figure S4. DSC thermogram of decyltriethylammonium bis(trifluoromethylsulfonyl)imide [TEA-C10][TFSI] collected in the heating/cooling rate of 10 K min-1.
   e. Figure S5. DSC thermogram of dodecyltriethylammonium bis(trifluoromethylsulfonyl)imide [TEA-C12][TFSI] collected in the heating/cooling rate of 10 K min-1.
   f. Figure S6. DSC thermogram of triethyltetradecylammonium bis(trifluoromethylsulfonyl)imide [TEA-C14][TFSI] collected in the heating/cooling rate of 10 K min-1.
   g. Figure S7. DSC thermogram of hexadecyltriethylammonium bis(trifluoromethylsulfonyl)imide [TEA-C16][TFSI] collected in the heating/cooling rate of 10 K min-1.
   h. Figure S8. FT-IR spectra of all prepared ILs
   i. Figure S9. Changes in the correlation distance of the observed SAXS peaks.
   j. Figure S10. Temperature-dependent self-diffusion coefficients and appropriate fits of [TEA-R][TFSI] ILs.
III. Additional Tables
   a. Table S1. Values of diffusion coefficient for each presented ionic liquid and for every measured temperature

I. Synthesis description and NMR Spectra of the prepared homologous series of alkyltriethylammonium bis(trifluoromethanesulfonyl)imides

Ionic liquids were prepared according to well-known literature protocols of (1) quaternization reaction and (2) ion-exchange reaction (Scheme 1). First, alkyltriethylammonium bromides were prepared in a reaction of triethylamine with an appropriate alkyl bromide (alkyl group from 4 to 16 carbon atoms) in a molar ratio of 1:1.1. The quaternization reaction according to S_N2 mechanism, takes place in acetonitrile, at elevated temperatures within 24 h. The reaction yields in every case exceed 80%. In the second step, an appropriate quaternary bromide was subjected to metathesis reaction with lithium bis(trifluoromethylsulfonyl)imide, molar ratio 1:1, in water environment at room temperature for 24h. After this time, second phase (the prepared IL) was visible. The reaction mixture was afterwards transferred to the separating funnel, and the organic phase was washed with deionized water in order to remove inorganic salt (lithium bromide). Finally, the appropriate pure product dissolved in methylene chloride was dried under the anhydrous sodium sulfate for 24 h. Finally, organic phase was filtered, and methylene chloride was removed with rotary evaporator. The obtained IL was afterwards dried for 48h in a vacuum desiccator. Each of the synthesized IL was liquid at room temperature. Prior to use, each IL was dried in a desiccator for at least 24 h.

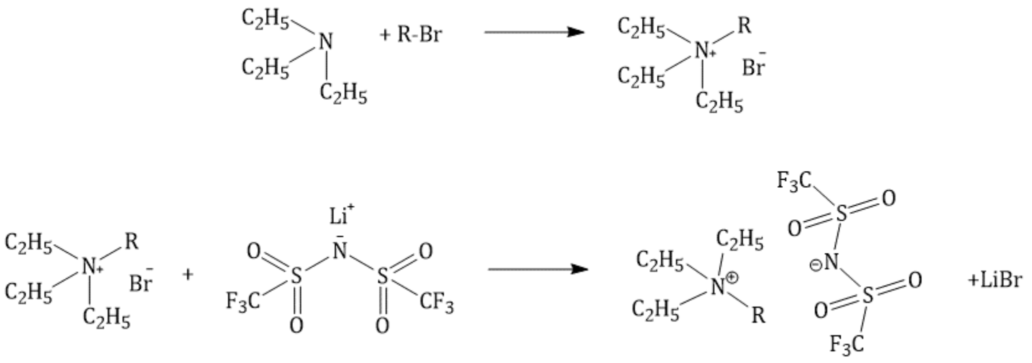

Scheme 1

^1H NMR spectra were recorded on an NMR Agilent spectrometer operating at 600 MHz.

Butyltriethylammonium bis(trifluoromethylsulfonyl)imide [TEA-C4][TFSI] (Chemical formula: $C_{12}H_{24}F_6N_2O_2S_2$, molecular weight: 438.45 g mol^{-1}) δH ($CDCl_3$, 296 K, 600 MHz): 0.90 (t, J = 7.2 Hz, 3H, N^+–$CH_2CH_2CH_2C\underline{H_3}$); 1.19-1.22 (m, 9H, N^+–$(CH_2C\underline{H_3})_3$); 1.28-1.32 (m, 2H, N^+–$CH_2CH_2C\underline{H_2}CH_3$); 1.48-1.56 (m, 2H, N^+–$CH_2C\underline{H_2}CH_2CH_3$); 2.96-3.02 (m, 2H, N^+–$C\underline{H_2}CH_2CH_2CH_3$); 3.16 (q, J = 6.9 Hz, 6H, N^+–$(C\underline{H_2}CH_3)_3$).

Triethylhexylammonium bis(trifluoromethylsulfonyl)imide [TEA-C6][TFSI] (Chemical formula: $C_{14}H_{28}F_6N_2O_2S_2$, molecular weight: 466.50 g mol^{-1}) δH ($CDCl_3$, 296 K, 600 MHz): 0.88 (t, 3H, N^+–$CH_2CH_2(CH_2)_3C\underline{H_3}$); 1.27-1.34 (m, 15H, N^+–$(CH_2C\underline{H_3})_3$), N^+–$CH_2CH_2(C\underline{H_2})_3CH_3$); 1.57-1.63 (m, 2H, N^+–$CH_2C\underline{H_2}(CH_2)_3CH_3$); 3.05-3.07 (m, 2H, N^+–$C\underline{H_2}CH_2(CH_2)_3CH_3$); 3.24 (q, J = 7.3 Hz, 6H, N^+–$(C\underline{H_2}CH_3)_3$).

Triethyloctylammonium bis(trifluoromethylsulfonyl)imide [TEA-C8][TFSI] (Chemical formula: $C_{16}H_{32}F_6N_2O_2S_2$, molecular weight: 494.56 g mol^{-1}) δH ($CDCl_3$, 296 K, 600 MHz): 0.88 (t, J = 8.1 Hz, 3H, N^+–$CH_2CH_2(CH_2)_5C\underline{H_3}$); 1.29-1.34 (m, 19H, N^+–$(CH_2C\underline{H_3})_3$), N^+–$CH_2CH_2(C\underline{H_2})_5CH_3$); 1.58-1.63 (m, 2H, N^+–$CH_2C\underline{H_2}(CH_2)_5CH_3$); 3.04-3.10 (m, 2H, N^+–$C\underline{H_2}CH_2(CH_2)_5CH_3$); 3.25 (q, J = 7.5 Hz, 6H, N^+–$(C\underline{H_2}CH_3)_3$).

Decyltriethylammonium bis(trifluoromethylsulfonyl)imide [TEA-C10][TFSI] (Chemical formula: $C_{18}H_{36}F_6N_2O_2S_2$, molecular weight: 522.61 g mol^{-1}) δH ($CDCl_3$, 296 K, 600 MHz): 0.87 (t, J=8.7 Hz, 3H, $N^+{-}CH_2CH_2(CH_2)_7C\underline{H_3}$); 1.25-1.35 (m, 23H, $N^+{-}(CH_2C\underline{H_3})_3$), $N^+{-}CH_2CH_2(C\underline{H_2})_7CH_3$); 1.60-1.63 (m, 2H, $N^+{-}CH_2C\underline{H_2}(CH_2)_7CH_3$); 3.04-3.10 (m, 2H, $N^+{-}C\underline{H_2}CH_2(CH_2)_7CH_3$); 3.25 (q, J = 9.0 Hz, 6H, $N^+{-}(C\underline{H_2}CH_3)_3$).

Dodecyltriethylammonium bis(trifluoromethylsulfonyl)imide [TEA-C12][TFSI] (Chemical formula: $C_{20}H_{40}F_6N_2O_2S_2$, molecular weight: 550.66 g mol^{-1}) δH ($CDCl_3$, 296 K, 600 MHz): 0.88 (t, J = 7.1 Hz, 3H, $N^+{-}CH_2CH_2(CH_2)_9C\underline{H_3}$); 1.26-1.35 (m, 27H, $N^+{-}(CH_2C\underline{H_3})_3$), $N^+{-}CH_2CH_2(C\underline{H_2})_9CH_3$); 1.60-1.63 (m, 2H, $N^+{-}CH_2C\underline{H_2}(CH_2)_9CH_3$); 3.04-3.10 (m, 2H, $N^+{-}C\underline{H_2}CH_2(CH_2)_9CH_3$); 3.26 (q, J = 7.8 Hz, 6H, $N^+{-}(C\underline{H_2}CH_3)_3$).

Triethyltetradecylammonium bis(trifluoromethylsulfonyl)imide [TEA-C14][TFSI] (Chemical formula: $C_{22}H_{44}F_6N_2O_2S_2$, molecular weight: 578.72 g mol^{-1}) δH ($CDCl_3$, 296 K, 600 MHz): 0.87 (t, J = 9.3 Hz, 3H, $N^+{-}CH_2CH_2(CH_2)_{11}C\underline{H_3}$); 1.24-1.34 (m, 31H, $N^+{-}(CH_2C\underline{H_3})_3$), $N^+{-}CH_2CH_2(C\underline{H_2})_{11}CH_3$); 1.59-1.63 (m, 2H, $N^+{-}CH_2C\underline{H_2}(CH_2)_{11}CH_3$); 3.04-3.10 (m, 2H, $N^+{-}C\underline{H_2}CH_2(CH_2)_{11}CH_3$); 3.25 (q, J = 9.4 Hz, 6H, $N^+{-}(C\underline{H_2}CH_3)_3$).

Hexadecyltriethylammonium bis(trifluoromethylsulfonyl)imide [TEA-C16][TFSI] (Chemical formula: $C_{24}H_{48}F_6N_2O_2S_2$, molecular weight: 606.77 g mol^{-1}) δH ($CDCl_3$, 296 K, 600 MHz): 0.88 (t, J = 6.9 Hz, 3H, $N^+{-}CH_2CH_2(CH_2)_{13}C\underline{H_3}$); 1.24-1.36 (m, 35H, $N^+{-}(CH_2C\underline{H_3})_3$), $N^+{-}CH_2CH_2(C\underline{H_2})_{13}CH_3$); 1.57-1.64 (m, 2H, $N^+{-}CH_2C\underline{H_2}(CH_2)_{13}CH_3$); 3.04-3.10 (m, 2H, $N^+{-}C\underline{H_2}CH_2(CH_2)_{13}CH_3$); 3.26 (q, J = 7.2 Hz, 6H, $N^+{-}(C\underline{H_2}CH_3)_3$).

II. Additional Figures:

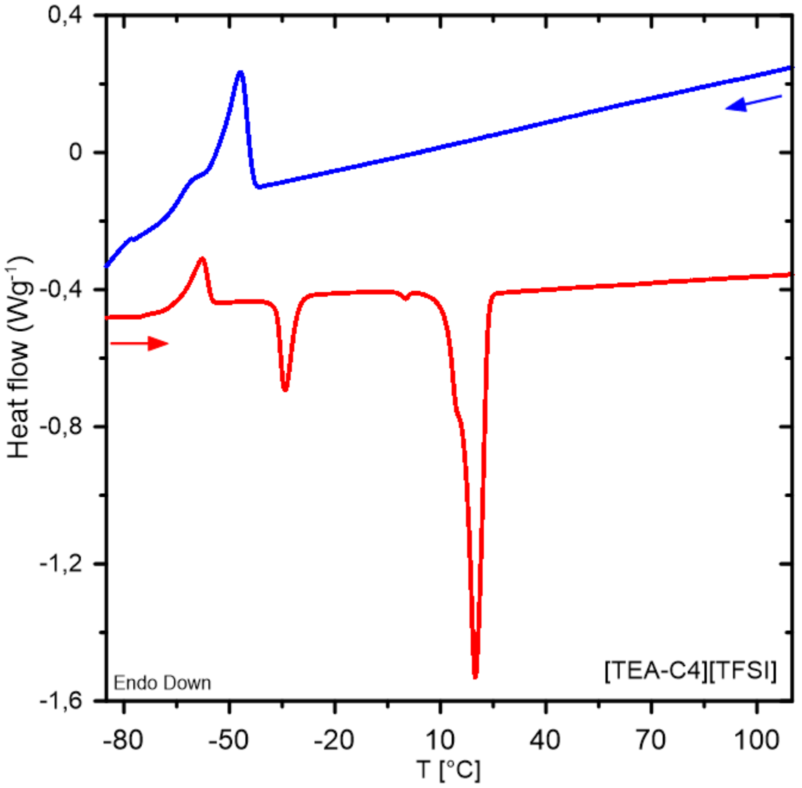

Figure S3. *DSC thermogram of butyltriethylammonium bis(trifluoromethylsulfonyl)imide [TEA-C4][TFSI] collected in the heating/cooling rate of 10 K min^{-1}.*

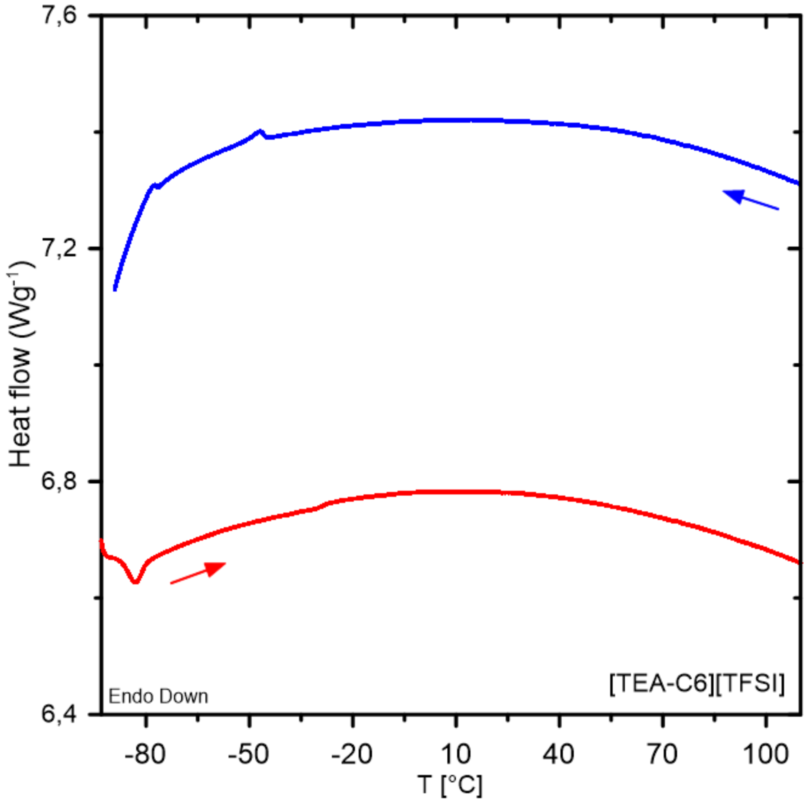

Figure S4. *DSC thermogram of triethylhexylammonium bis(trifluoromethylsulfonyl)imide [TEA-C6][TFSI] collected in the heating/cooling rate of 10 K min^{-1}.*

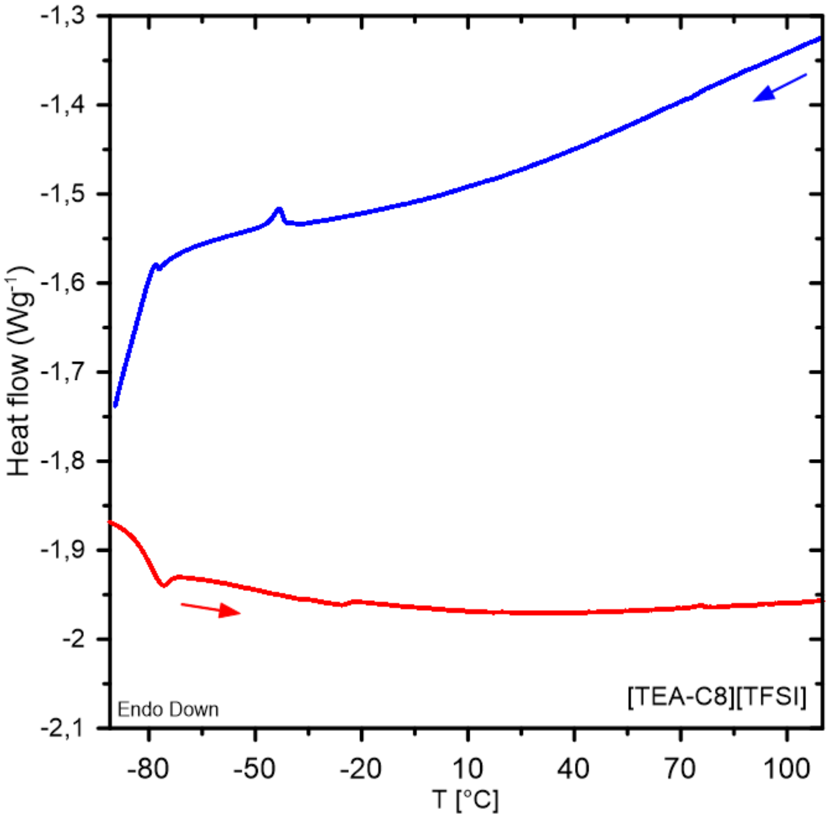

Figure S5. *DSC thermogram of triethyloctylammonium bis(trifluoromethylsulfonyl)imide [TEA-C8][TFSI] collected in the heating/cooling rate of 10 K min*$^{-1}$.

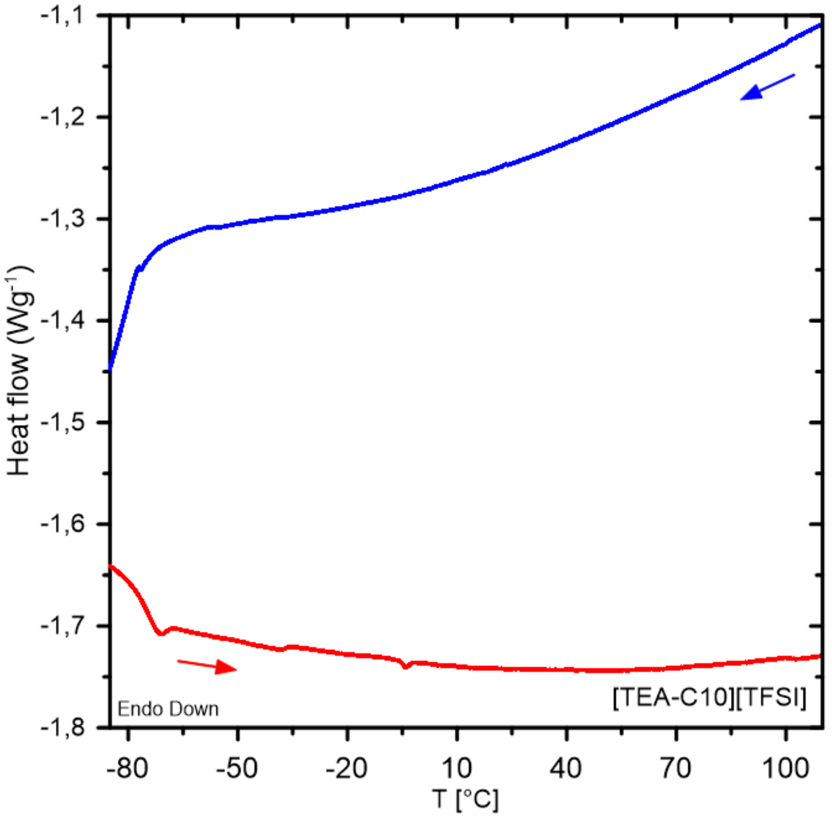

Figure S6. *DSC thermogram of decyltriethylammonium bis(trifluoromethylsulfonyl)imide [TEA-C10][TFSI] collected in the heating/cooling rate of 10 K min*$^{-1}$.

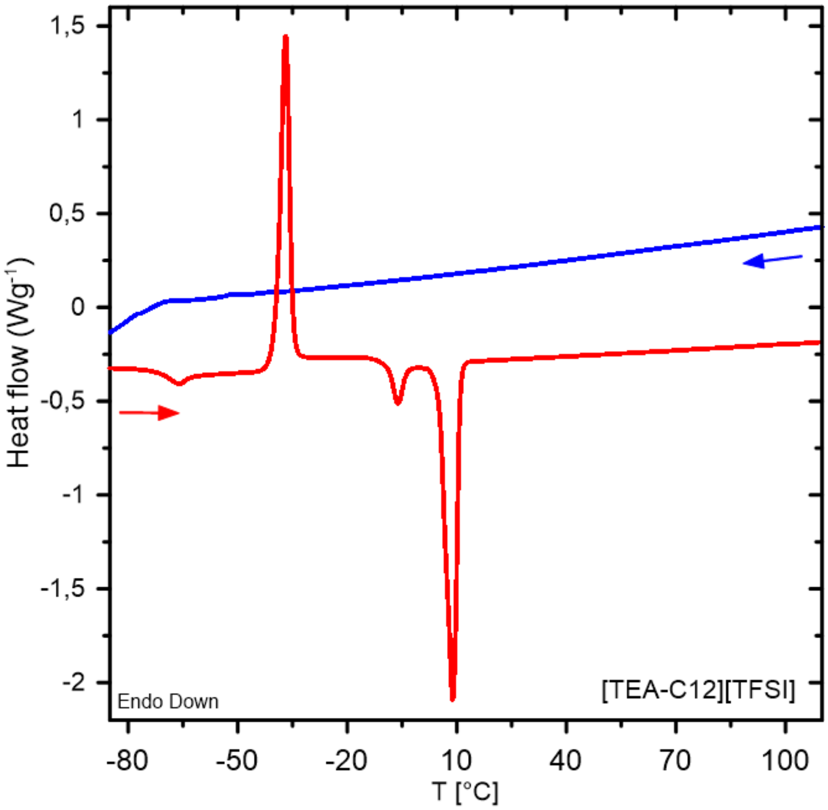

Figure S7. *DSC thermogram of dodecyltriethylammonium bis(trifluoromethylsulfonyl)imide [TEA-C12][TFSI] collected in the heating/cooling rate of 10 K min-1.*

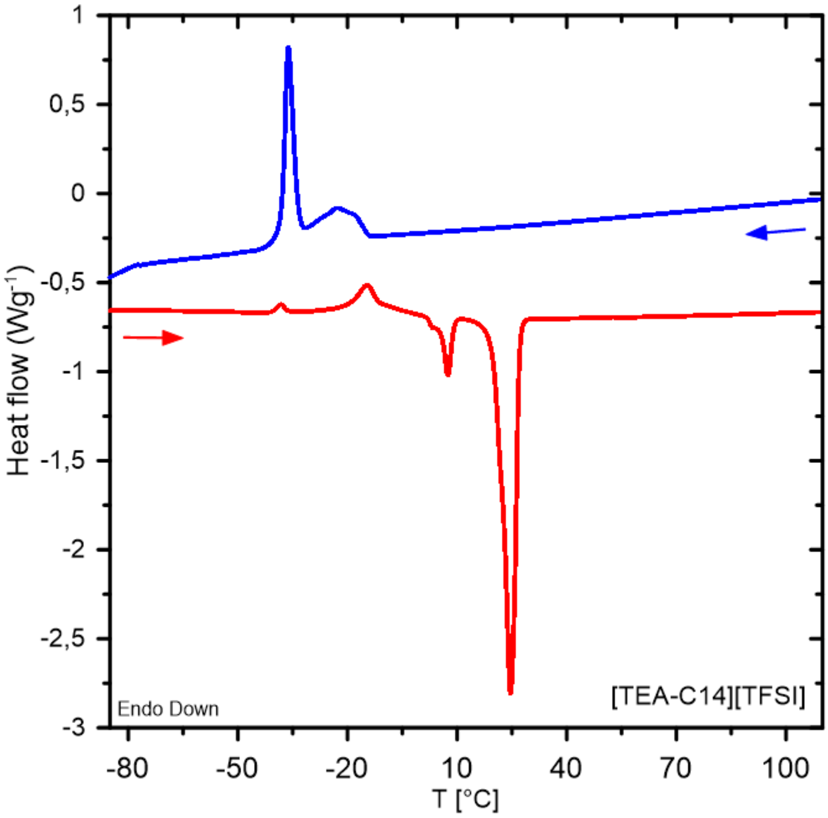

Figure S8. *DSC thermogram of triethyltetradecylammonium bis(trifluoromethylsulfonyl)imide [TEA-C14][TFSI] collected in the heating/cooling rate of 10 K min*$^{-1}$.

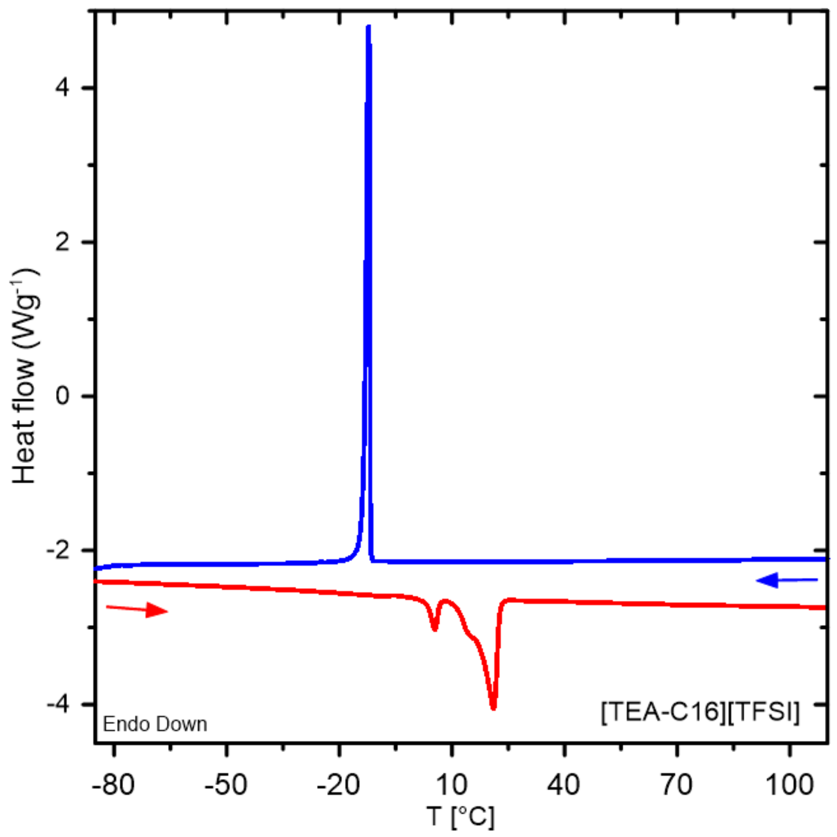

Figure S9. *DSC thermogram of hexadecyltriethylammonium bis(trifluoromethylsulfonyl)imide [TEA-C16][TFSI] collected in the heating/cooling rate of 10 K min^{-1}.*

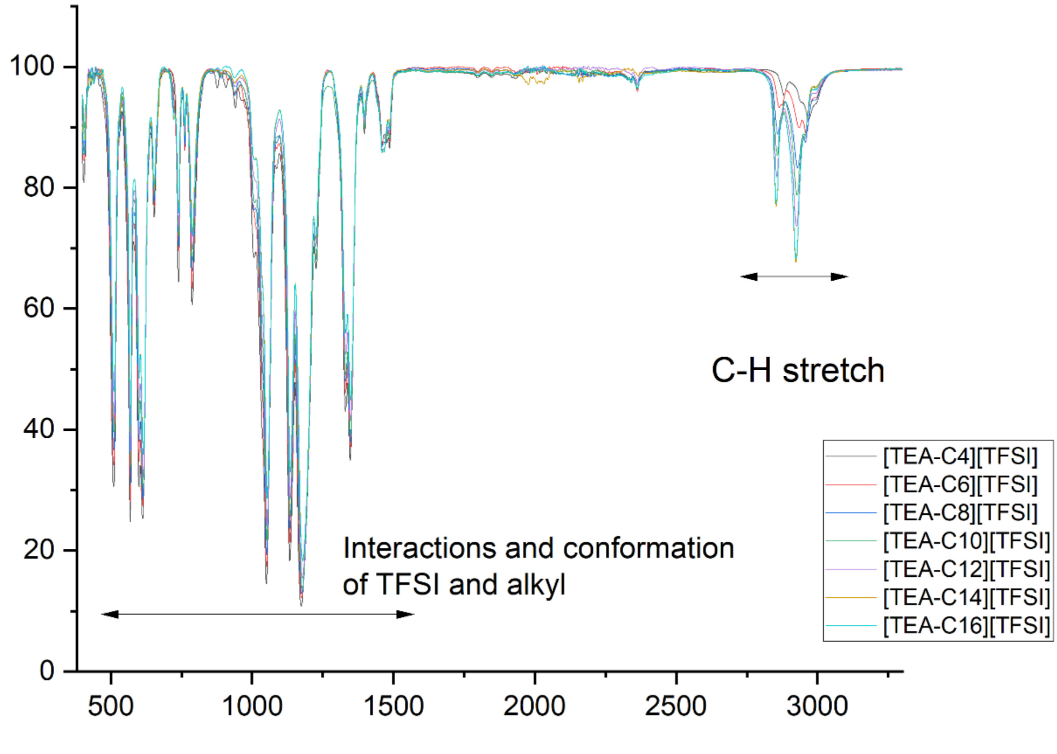

Figure S8. *FT-IR spectra of all prepared ILs*

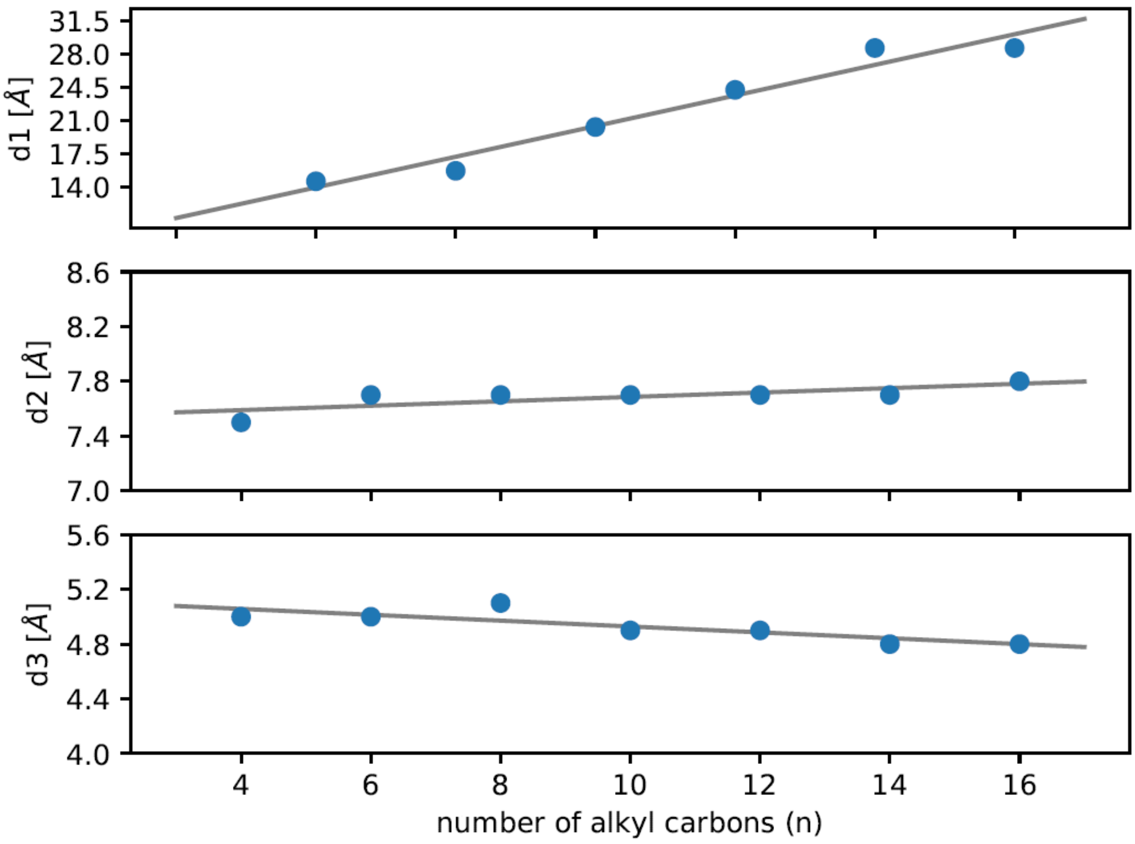

Figure S9. *Changes in the correlation distance of the observed SAXS peaks (d1, d2, and d3 respectively)*

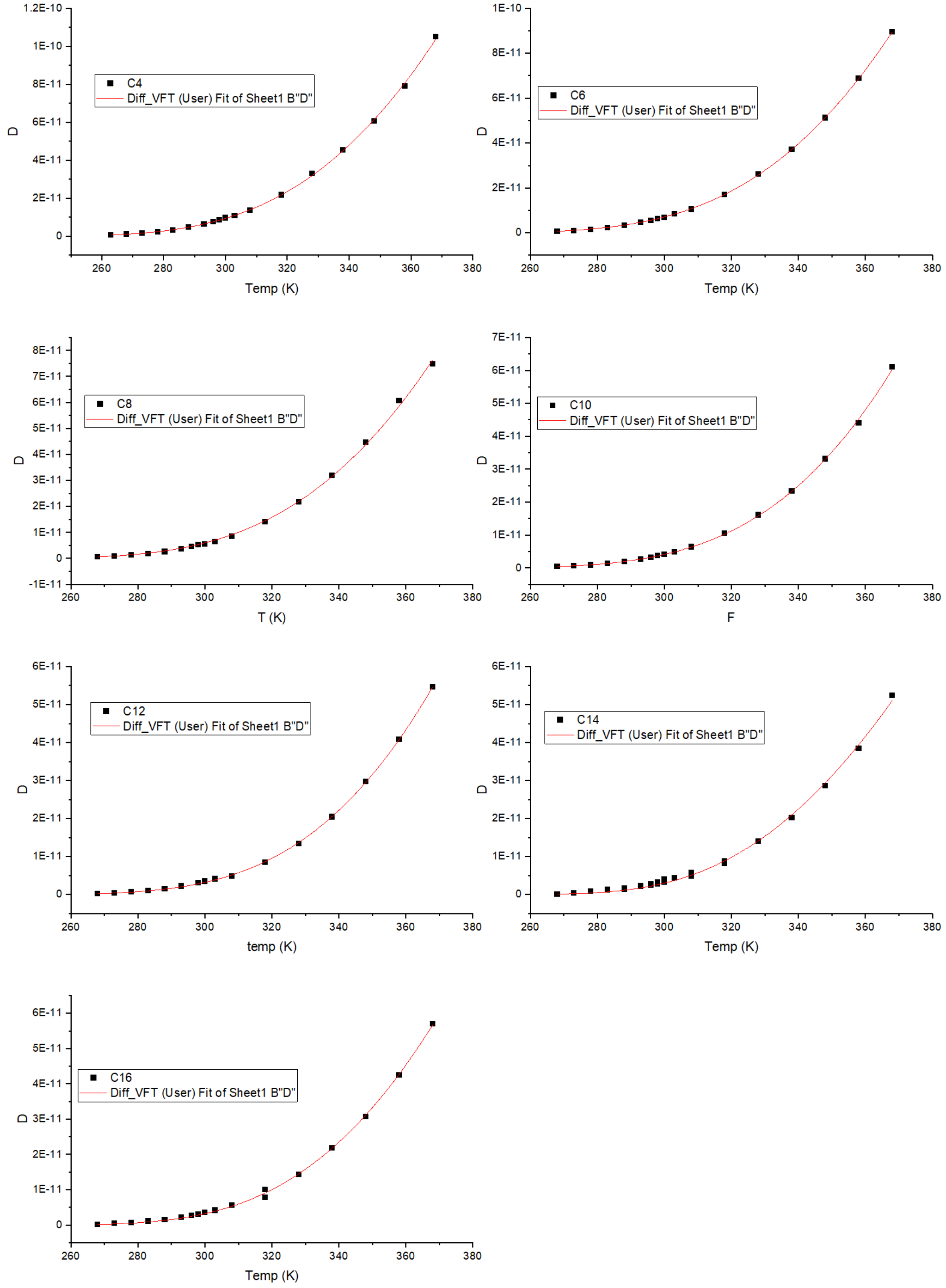

Figure S10. *Temperature-dependent self-diffusion coefficients and appropriate fits of [TEA-R][TFSI] ILs.*

III. Additional Tables

Table S1. Values of diffusion coefficient for each presented ionic liquid and for every measured temperature

Temperature [ºC]	Diffusion coefficient [m^2/s]						
	$TEAC_4$	$TEAC_6$	$TEAC_8$	$TEAC_{10}$	$TEAC_{12}$	$TEAC_{14}$	$TEAC_{16}$
**95**	1.05 e-10	8.95 e-11	7.47 e-11	6.10 e-11	5.47 e-11	5.24 e-11	5.71 e-11
**85**	7.91 e-11	6.88 e-11	6.07 e-11	4.40 e-11	4.08 e-11	3.84 e-11	4.26 e-11
**75**	6.07 e-11	5.13 e-11	4.47 e-11	3.31 e-11	2.97 e-11	2.87 e-11	3.08 e-11
**65**	4.53 e-11	3.72 e-11	3.19 e-11	2.33 e-11	2.05 e-11	2.02 e-11	2.18 e-11
**55**	3.32 e-11	2.61 e-11	2.16 e-11	1.61 e-11	1.34 e-11	1.40 e-11	1.43 e-11
**45**	2.18 e-11	1.71 e-11	1.41 e-11	1.05 e-11	8.40 e-12	8.15 e-12	7.86 e-12
**35**	1.38 e-11	1.05 e-11	8.52 e-12	6.41 e-12	4.90 e-12	5.73 e-12	5.66 e-12
**30**	1.09 e-11	8.50 e-12	6.49 e-12	4.88 e-12	4.10 e-12	4.37 e-12	4.12 e-12
**27**	9.74 e-12	6.77 e-12	5.47 e-12	4.14 e-12	3.45 e-12	3.30 e-12	3.50 e-12
**25**	8.59 e-12	6.28 e-12	5.17 e-12	3.77 e-12	3.05 e-12	2.85 e-12	3.03 e-12
**23**	7.60 e-12	5.57 e-12	4.51 e-12	3.28 e-12	-	2.50 e-12	2.66 e-12
**20**	6.45 e-12	4.64 e-12	3.65 e-12	2.68 e-12	2.23 e-12	2.14 e-12	2.16 e-12
**15**	4.72 e-12	3.27 e-12	2.58 e-12	1.92 e-12	1.54 e-12	1.70 e-12	1.50 e-12
**10**	3.33 e-12	2.25 e-12	1.81 e-12	1.33 e-12	1.07 e-12	1.16 e-12	1.07 e-12
**5**	2.27 e-12	1.51 e-12	1.26 e-12	9.27 e-13	6.95 e-13	8.10 e-13	6.89 e-13
**0**	1.57 e-12	1.03 e-12	8.10 e-13	6.14 e-13	4.09 e-13	4.58 e-13	4.22 e-13
**-5**	1.10 e-12	7.20 e-13	5.15 e-13	4.33 e-13	2.52 e-13	9.55 e-14	1.75 e-13
